# ID 283 - Avaliação da Aderência ao Protocolo Clínico e Diretrizes Terapêuticas (PCDT) para Mucopolissacaridose Tipo 2 (MPS2): comparação entre prática clínica real e dados de *Time-Driven Activity-Based Costing* (TDABC) na Policlínica Codajás/AM no Contexto da Jorna

**DOI:** 10.5327/2237-9622.2025.v34s1.283

**Published:** 2025-11-25

**Authors:** Myrianne Gilsara Soares e Barbosa, Camila Azevedo, Vânia Mesquita Gadelha Prazeres, Sabrina Macely Souza dos Santos, Dhallya Andressa da Silva Cruz, Marcelo Nita, Luana Lopes, Thiago Godoy, Têmis Félix

**Keywords:** *Time-Driven Activity-Based Costing* (TDABC), mucopolissacaridose tipo 2, custeio, jornada de valor

## Abstract

**Introdução:**

O Protocolo Clínico e Diretrizes Terapêuticas (PCDT) é um guia técnico que estabelece critérios baseados em evidências científicas para o diagnóstico, tratamento e acompanhamento de uma doença ou condição de saúde específica. O propósito deste estudo foi mapear as jornadas assistenciais de pacientes com MPS2 na Policlínica Codajás, localizada no Amazonas, e compará-las com o PCDT estabelecido pelo Ministério da Saúde (MS), permitindo, dessa forma, avaliar a aderência, identificar divergências e propor melhorias no atendimento.

**Método:**

O estudo "Jornada Assistencial de Valor para Pacientes com Doenças Raras (JAV-RARAS)" é uma investigação prospectiva de alcance nacional que emprega a metodologia (TDABC) para avaliar os processos e custos associados ao manejo de 21 doenças raras. O estudo identificou os custos reais anuais do manejo de pacientes com MPS2, praticados pelas organizações de saúde no Sistema Único de Saúde (SUS), por meio de entrevistas com profissionais de saúde e análises de processos administrativos e assistenciais nos centros participantes. O custo definido pelo PCDT foi calculado com base nas definições contidas nele, permitindo uma comparação direta com a prática clínica atual.

**Resultados:**

Os resultados preliminares indicam que, na prática real, o custo médio anual do centro foi de R$ 832.180,22, enquanto o custo baseado no PCDT foi de R$ 343.677,50. Foram observadas diferenças significativas nos custos entre os tratamentos propostos no PCDT e os tratamentos praticados na vida real. De fato, o custo médio estimado no PCDT para o tratamento foi de R$ 342.292,80, enquanto o custo real praticado pela Policlínica Codajás foi de R$ 827.485,44.

**Conclusão:**

A análise preliminar dos custos revelou que a prática clínica atual em centros médicos é mais dispendiosa do que a definida no PCDT para o tratamento da MPS2. A discrepância observada entre os custos sugere que as necessidades específicas dos pacientes com MPS2 podem não estar sendo completamente atendidas pelo PCDT atual. Esse resultado ressalta a importância de manter os protocolos clínicos atualizados, por meio do monitoramento em tempo real da aderência a eles, e adaptá-los às demandas dos pacientes, garantindo assim uma assistência médica de qualidade e efetiva.

